# Supplementary material for: The interactome of intact mitochondria by cross-linking mass spectrometry provides evidence for coexisting respiratory supercomplexes
Source: Mol Cell Proteomics. 2017 Dec 8;17(2):216–32. doi: 10.1074/mcp.RA117.000470 (PMC5795388; doi:10.1074/mcp.RA117.000470)
Supplement: Supplemental Data [file supp_RA117.000470_133922_0_supp_23978_fzffwf.zip › spectra_annotation/mito_DR_spectra_annotation/296-1-38-1-38-1.pdf]

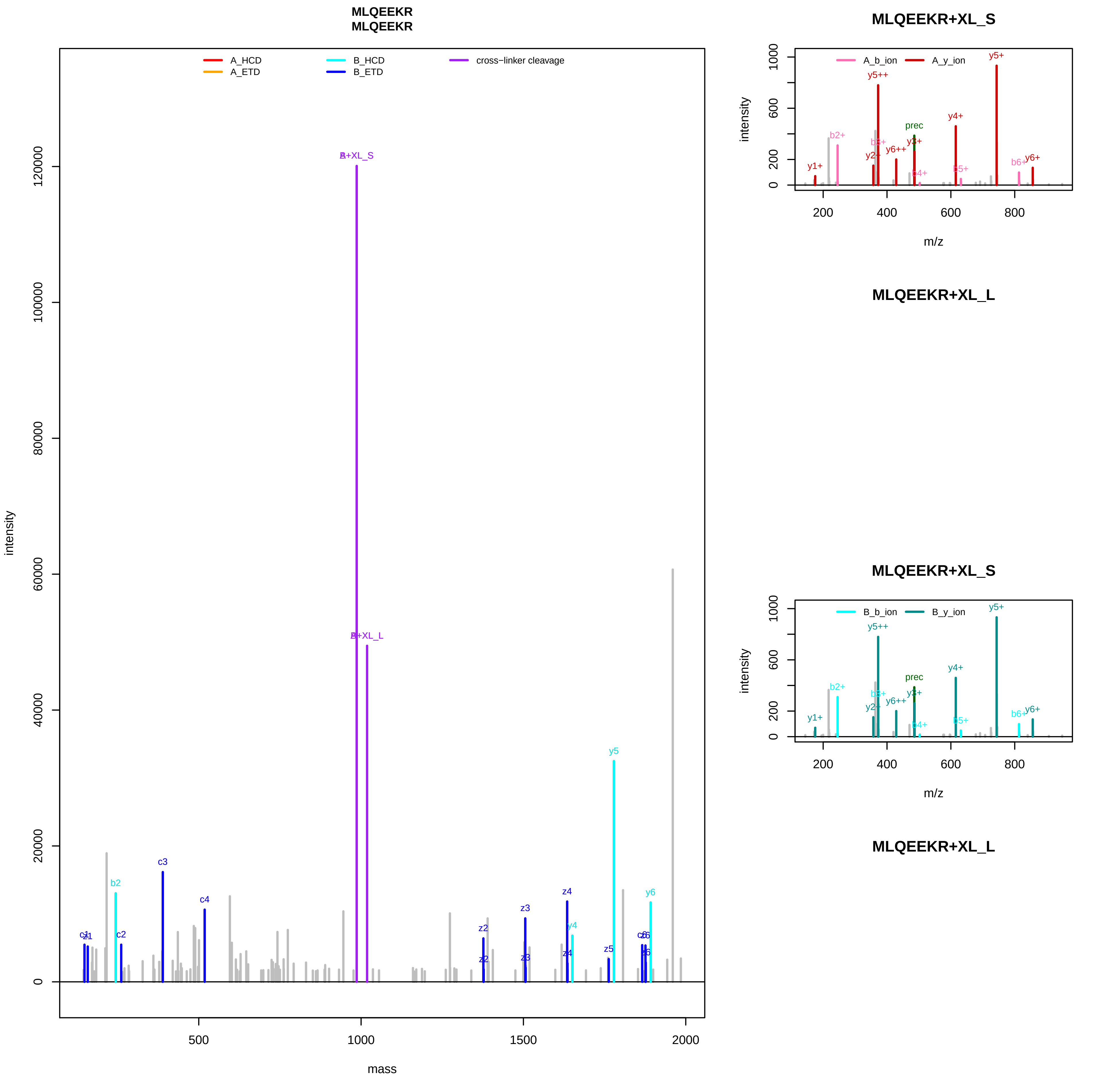

MLQEEKR
MLQEEKR
A_HCD
A_ETD
B_HCD
B_ETD
cross−linker cleavage
B+XL_S
B+XL_L
c1
z1
b2
c2
c3
c4
z2
z3
z4
y4
z5
y5
c6
z6
y6
intensity
mass
0
20000
40000
60000
80000
100000
120000
500
1000
1500
2000
MLQEEKR+XL_S
A_b_ion
A_y_ion
y1+
b2+
y2+
b3+
y5++
prec
y3+
y6++
b4+
y4+
b5+
y5+
b6+
y6+
intensity
m/z
MLQEEKR+XL_L
MLQEEKR+XL_S
B_b_ion
B_y_ion
MLQEEKR+XL_L
0
200
600
1000
400
800
